# Production of recombinant proteins in *E. coli *by the heat inducible expression system based on the phage lambda pL and/or pR promoters

**DOI:** 10.1186/1475-2859-9-18

**Published:** 2010-03-19

**Authors:** Norma A Valdez-Cruz, Luis Caspeta, Néstor O Pérez, Octavio T Ramírez, Mauricio A Trujillo-Roldán

**Affiliations:** 1 Departamento de Medicina Molecular y Bioprocesos, Instituto de Biotecnología, Universidad Nacional Autónoma de México. Avenida Universidad 2001, Cuernavaca Morelos, México; 2 Probiomed S.A. de C.V. Planta Tenancingo, Cruce de Carreteras Acatzingo-Zumpahuacan SN, C.P. 52400 Tenancingo, Edo. de México, México; 3 Unidad de Bioprocesos, Departamento de Inmunología, Instituto de Investigaciones Biomédicas, Universidad Nacional Autónoma de México, A.P. 70228, México, D.F., C.P. 04510, México

## Abstract

The temperature inducible expression system, based on the pL and/or pR phage lambda promoters regulated by the thermolabile cI857 repressor has been widely use to produce recombinant proteins in prokariotic cells. In this expression system, induction of heterologous protein is achieved by increasing the culture temperature, generally above 37°C. Concomitant to the overexpression of heterologous protein, the increase in temperature also causes a variety of complex stress responses. Many studies have reported the use of such temperature inducible expression system, however only few discuss the simultaneous stress effects caused by recombinant protein production and the up-shift in temperature. Understanding the integral effect of such responses should be useful to develop improved strategies for high yield protein production and recovery. Here, we describe the current status of the heat inducible expression system based on the pL and/or pR λ phage promoters, focusing on recent developments on expression vehicles, the stress responses at the molecular and physiological level that occur after heat induction, and bioprocessing factors that affect protein overexpression, including culture operation variables and induction strategies.

## Heat induction: A compromise between recombinant protein production and stress

*E. coli *expression systems have been the preferred option for producing many recombinant proteins in high quantities and low production costs [1-6]. Among the underlying reasons for such a widespread use of *E. coli *is the availability of a variety of strong inducible promoters [7]. The promoters commonly employed for heterologous protein expression require the addition of an inducer molecule, the depletion or addition of a nutrient, or a shift in a physical or physicochemical factor, such as pH [4]. Yet, each option can present different shortcomings. For instance chemical inducers, such as IPTG and antibiotics, can be expensive and toxic, and their presence in either the final product or in the waste effluents of the bioprocess represent a hazard that must be eliminated [8,9]. Accordingly, additional controls and downstream operations may be required to remove chemical inducers, particularly from pharmaceutical-grade proteins and products intended for human use, complicating the bioprocess and increasing its cost [10]. In systems based on nutrient exhaustion, such as depletion of an amino acid from the culture broth, starvation can affect cell metabolism or synthesis of the recombinant protein [11,12] and a precise control of the induction timing is difficult. In the case of pH-inducible expression systems, few vectors are available, characterization studies are still insufficient, and pH for induction can depart from the optimal pH for physiological conditions [13]. Many of the drawbacks mentioned can be overcome when using the thermo-regulated expression system reviewed here.

The thermo-regulated expression system has been successfully used for the production of many recombinant proteins and peptides since it relies on a strong and finely regulated promoter, and the use of special media, toxic or expensive chemical inducers is avoided [14]. Furthermore, culture handling and contamination risks are minimized, as temperature in fermentors can be readily modified by external means. All these features are relevant conditions when producing therapeutic recombinant proteins. In addition, the system is easily scalable, although heat transfer limitations of large-scale bioreactors should be taken in consideration, as heating rate will decrease as culture volume increases [15].

**Figure 1:**
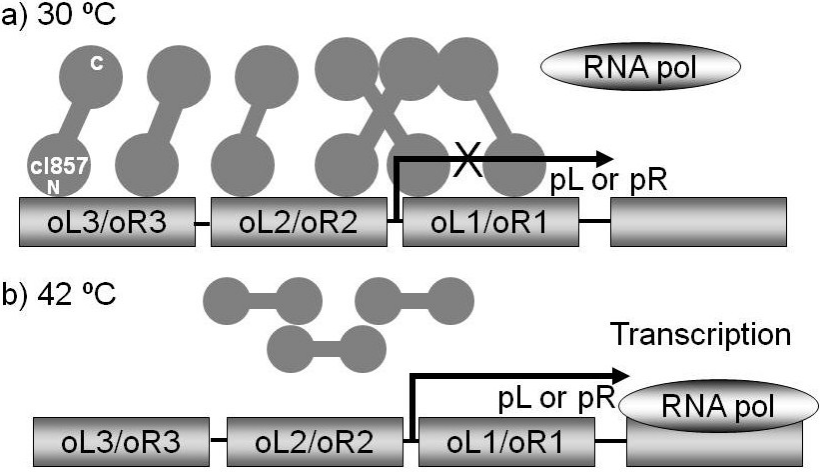
**Representation of the pL/pR promoters controlled by the cI857 repressor**. The cI857 interacts with three operator domains (oL3/oR3, oL2/oR2, and oL1/oR1), repressing transcription. The heterologous gene is localized downstream from the pL or pR promoters. a) The cI857 forms dimers that bind to the oL or oR region and block transcription by RNA polymerase. b) The interaction of cI857 with oL or oR regions, is released up to 37°C, permitting transcription by RNA polymerase.

The thermo-regulated expression system is based on the insertion of the gene of interest into different vectors containing the strong major leftward (pL) and/or rightward (pR) promoters. The gene cloned downstream of the λ promoters can then be efficiently regulated by the mutant thermolabile cI857 repressor of bacteriophage λ[10,16]. Gene expression is inhibited at culture temperatures below 37°C (normally in the range of 28-32°C) whereas transcription by the host RNA polymerase ensues upon inactivation of the mutant repressor by increasing the temperature above 37°C (Figure 1) [16]. Similarly to what occurs in all other expression systems, the production of heterologous protein causes important stresses and metabolic unbalances. For instance, the overproduction of recombinant protein can trigger the heat shock like response, stringent response and the SOS response, and can result in a metabolic burden to the cells [17,18]. As a consequence, specific grow rate will in general decrease, and ribosome degradation [19] and alterations in the central carbon metabolism [20] can occur. Altogether, these effects can alter the quantity and quality of the foreign protein produced [18,21,22]. However, in contrast to other expression systems, heat induction also triggers the heat-shock response (HSR) that is controlled by the alternative sigma factor σ32 (*rpoH *gene product) [23,24]. The HSR includes a rapid and selective synthesis of heat-shock proteins (hsp) soon after temperature increases. Thereafter, an adaptation period occurs with a lower rate of protein synthesis that latter reaches a new steady-state level. The hsp serve as chaperones and proteases involved in folding, degradation, and proper feedback regulation of the HSR [24,25]. In addition to the synthesis of hsp, the physiological response of *E. coli *after a heat shock also includes the temporary decrease in growth rate and changes in cell membranes due to modification of the ratio of lipids and integral membrane proteins [26].

Many reports exist of recombinant protein production under the heat-inducible system, and many other separate studies also exist of the HSR. Nonetheless, activation and regulation of the HSR in a thermoinduced recombinant protein productive system has been scarcely studied. This review focuses on the regulation of the inducible expression λpL/pR-cI857 system, describing the molecular and physiological changes on the host cells caused by temperature associated stresses during thermoinduction, and the relation of such effects with the productivity and quality of the recombinant proteins.

## Molecular Biology of λpL/pR-cI857

Lambda phage elements like those that comprise the plasmids λpL/pR-cI857 and their regulation have been used for the production of foreign products in *E coli *[27].

### Lambda phage and the important elements for the λpL/pR expression system

The lambda phage is a dsDNA virus that infects *E. coli *and presents two lifecycle pathways [28]. The first, called the lytic pathway, consists of infection, rapid replication, assembling, and release of viral particles that can start a new cycle by infecting other hosts [27]. The lytic cycle seems to be favored when the host is healthy. The second is the lysogenic pathway, in which the lambda phage DNA is inserted into the host chromosome and is replicated with the rest of the bacterial DNA, remaining inactive in latent form [27,29]. The λ phage circular genome is formed by 48,502 base pairs that code for lytic genes, including the early lytic genes expressed from the pL and pR promoters, the late lytic genes under the pR' promoter, and the lysogenic genes expressed under the promoter for repressor maintenance (pRM) [27].

Gene regulation in bacteriophage λ is controlled by a key component, the λcI repressor that acts as a genetic switch between the lysogenic growth and lytic development [30,31]. cI is a protein formed by 236 amino acids distributed in two domains. In a lysogenic state, the host is not lysed because cI repressor prevents transcription of λ lytic genes. During lysogenic growth, the cI dimer repressor binds in a cooperative form, by protein-protein interactions of their C-terminal domains [32,33], to the operator regions oR (oR1 and oR2) and oL (oL1, oL2, and oL3), blocking the pR and pL promoters, respectively [34]. Moreover, cI binding allows the transcription from pRM in a concentration-dependent manner [29,31,35]. cI can also repress pRM in a cooperative manner by binding to oR3 after oL3 and oR3 are juxtaposed during DNA loop formation mediated by octamerization of cI dimers on oR1, oR2, oL1, and oL2 [34]. However, cI concentration must be 5-times higher than that found normally in the lysogenic state to occupy 50% of oR3, otherwise, occupancy in the lysogen is less than 20% [36,37]. Accordingly, λcI repressor has little negative autoregulation and physiological effects in the lysogenic state [37,38]. The presence of cI alone can maintain the lysogenic state, however a switch to the lytic pathway occurs when the host SOS response is triggered by DNA damage, which in turn induces the expression of the protein Rec-A that activates cI autocleavage [39-41].

### Regulation of the λpL/pR-cI857 system by temperature or other inducers

In 1966 Margaret Lieb identified λ lytic temperature sensitive (Ts) mutants that were induced by temperature up-shifts [42]. One of these mutants is cI857, which has the substitution of Ala66 by Thr in the amino-terminal region of the repressor. This mutant retains wild-type properties at low temperature, but is unstable when temperature is elevated [43]. Such finding opened the possibility for physically regulating the tight control switch of cI binding affinity to λpL/pR promoters and constituted the foundation for its biotechnological exploitation within expression systems for heterologous protein production in *E. coli *(Figure 1). The recombinant proteins initially expressed in the λpL/pR-cI857 system were those from *E. coli *[44-47], phages [48,49], and virus [50]. Soon after, the attention for this expression system broaden to other proteins of biotechnological interest, in particular for the production of human therapeutic recombinant protein such as β interferon, insulin, recombinant human growth hormone (rHGH), and tumor necrosis factor [7,10,51-55] (Table 1).

**Table 1:** Summary of reported yields obtained with the λpL/pR/cI857 thermoinduced expression system.

Protein	Plasmid	Culture conditions; induction strategy	Production	System	**Ref**.
**Recombinant protein localized in cytoplasm**					

UVRA	pGHY5003	BC; 30 to 42°C	7% TCP	λpl, cI857	[44]
IFN-β	pPLc245-HFIF25	BC; 28 to 42°C	4% TCP	λpL/cI857	[61]
GXP	pHEGPT	BC, SF; 30 to 42°C	5% TCP	λpL/cI857	[58]
IHF α, β	pPlhima-1, pPLhipohim A-s	BC; 31 to 42°C	3 mg/L	λpL/cI857	[169]
rpGH	p-LARpGH	BC, SF; 30 to 42°C (3-4 h), temp reached in 5-8 min	15% TCP	λpL/lcI857	[170]
rpGH	p-LARpGH	FB; 30 to 42°C	20% TCP	λpL/lcI857	[170]
β-gal	pJLACZ	CC; 28 to 40°C	3,037 U/ml/OD	λpL cI857	[16]
β-gal	pJLACZ	CC; 28 to 42°C	7,327 U/ml/OD	λpL cI857	[16]
SpA-β-gal		BC; 30 to 40°C	6.2 g/L	λpR	[65]
SpA-β-gal		FB; 30 to 40°C	19.2 g/L	λpR	[65]
β-gal	pACYC177; pRSET- lacZ	BC, SF; 30 to 42°C (2 min), reducing to 37°C	23500 U	λpL/cI857	[171]
β-gal	pACYC177; pRSET- lacZ	BC, SF- FB; 30 to 42°C (2 min), reducing to 37°C	23000 U	λpL/cI857	[171]
β-gal	pACYC177; pRSET- lacZ	FB; 30 to 42°C (2 min), reducing to 37°C	285000 U (0.95 g/L)	λpL/cI857	[171]
Carbamoylase	pTAH10; pT-GroE	BC, SF; 30 to 39°C reached abruptly (10 to 20 min), decrease to 37°C	7 U (0.14 U/mL)	λpLpR, cI857-T7 RNA pol	[67]
Carbamoylase	pTAH10; pT-GroE	BC; 30-39°C (10 to 20 min), decrease to 37°C	1830 U (1.2 U/mL)	λpLpR, cI857-T7 RNA pol	[67]
Carbamoylase	pTAH10; pT-GroE	FB; 30 to 39°C (10 min), decrease to 37°C	14256 U (5.8 U/mL)	λpLpR, cI857-T7 RNA pol	[67]
TNF-α	pCY-TNF	BC, SF; 30 to 42°C	12% soluble TCP	λpRpL cI 857	[10]
TNF-α	pCY-TNF	BC, 30 to 42°C (6 h)	11% soluble TCP	λpRPL cI 857	[10]
TRAIL	pBV-Trail	FB; 30 to 42°C (4 h)	1.4 g/L	Temperature inducible	[172]
GFP	pND-GFP	BC; 30 to 42°C (1 h), reducing to 30°C	30 mg/L	λpL/cI857	[137]
GFP	pND-GFP	BC; 37 to 42°C (1 h), reducing to 37°C	50 mg/L	λpL/cI857	[137]
GFP	pND-GFP	BC, SF; 37 to 42°C (1 h), reducing to 37°C	7 mg/L	λpL/cI857	[137]
GFP	pND-GFP	BC, SF; 37 to 42°C (1 h), reduction to 37°C (1 h), increasing to 42°C (1 h), reducing to 37°C	68 mg/L	λpL/cI857	[137]
GFP	pND-GFP	BC, SF; 30 to 42°C (1 h), reduction to 30°C (1 h), increase to 42°C (1 h), decrease to 30°C	45 mg/L	λpL/cI857	[137]
GFP	pND-GFP	FB; 30 to 42°C (30 min), decrease to 30°C, increase to 42°C (30 min), decrease to 30°C	273 mg/L	λpL/cI857	[137]

**IB localized in cytoplasm**					

IFN-γ	pPL-l	BC, SF; 28°C-42°C	0.3 g/L	λpL/cI857	[52]
IFN-γ	pPL-l	FB; 28 to 42°C	7.43 g/L	λpL/cI857	[52]
Insulin A- chain-Mut3sY	PMYW-A	BC, SF: BL21; 30 to 42°C	30% TCP	λpl/cI857	[53]
Insulin A- chain-Mut3sY	PMYW-A	FB, HCDC; 30 to 42°C	13% TCP	λpl, lcI857	[53]
Insulin B- chain- Mut3sY	PMYW-B	FB, HCDC; 30 to 42°C	18% TCP (4.6 g/L)	λpl, lcI857	[53]
IFN-α	pMYW-a	FB; 30 to 42°C	4 g/L	λpl, lcI857	[7]
rhBMP-2	pCYTEXP3-BMP-2	BC, SF 30 to 42°C	25% TCP	λpL, cI857	[94]
rhBMP-2	pCYTEXP3-BMP-2	FB; 30 to 42°C	20% TCP (8.5 g/L)	λpL, cI857	[94]
hFGF-2	plFGFB	FB; 30 to 42°C (6-8 h)	5-6 g/L	λpRPL cI857	[95]
hGH	pET21-hgh	FB (complex media); 30 to 42°C (30 min), reduction to 37°C (4 h)	11% TCP (2.0 g/L)	pET21-hgh; pGP1-2 gene 1 of T7 under λpL	[54]
hGH	pET21-hgh	FB (glycerol); 30 to 42°C (30 min), reduction to 37°C (4 h)	15% TCP (2.7 g/L)	pET21-hgh; pGP1-2 gene 1 of T7 under λpL	[54]
IFN-α 2b	pRSET-INFα 2b; pGP1-2	BC, SF; 30 to 42°C (5, 10 and 15 min)	650 mg/L	λPL, T7 RNA pol, cI857	[55]
His-tag-hPPI	λ-PL-cI857 pUC	FB; 30 to 42°C heat rate 0.4°C/min	3.3 g/L	λpL/cI857	[15]
His-tag- hPPI	λ-PL-cI857 pUC	FB; 30 to 42°C heat rate 0.8°C/min	2.2 g/L	λpL/cI857	[15]
His tag- hPPI	λ-PL-cI857 pUC	FB; 30 to 42°C heat rate 1.7°C/min	1.8 g/L	λpL/cI857	[15]
His tag- hPPI	λ-PL-cI857 pUC	FB; 30 to 42°C heat rate 6°C/min	1.9 g/L	λpL/cI857	[15]

**Recombinant protein localized in cytoplasm and supernatant**					

TK	pHETK2	BC, SF; 30°C to 42°C (16 h)	4% soluble TCP	λpL/cI857	[50]
TK	pHETK2	BC, SF; 30°C to 42°C (16 h)	4% soluble TCP	λpL/cI857	[50]
β-gal	pRA-A1Its-187Z	BC, SF; 30 to 40°C (5 h)	30% TCP (2.0 g/L)	λpR, cI857, PA1	[66]
β-gal	pRA-A1Its-187Z	BC; 30 to 40°C	22% TCP (2.0 g/L)	λpR, cI857, PA1	[66]
scFv	pCMT2b-scFv;pRcd1	BC, SF; 30°C to 42°C (7 h)	37 mg/L	λpL/cI857; Rcd lPR	[173]
scFv	pCMT2b-scFv;pRcd1	FB; 30 to 42°C (8 h)	160 mg/L	λpL/cI857; Rcd IPR	[173]

**Recombinant protein localized in supernatant**					

Glucagon-SEAP	pBLGlu-2	BC, SF; 30 to 40°C	3.4 mg/L/OD600	λpL/cI857	[174]

**Recombinant protein localized in periplasm**					

hGH-fusion to DsbA	DsbA-hGH	BC, SF; 30 to 42°C	19%	λpL/cI857	[125]
hGH	lPL-DsbA-hGH/pRK248cIts	FB; 30 to 42°C heat rate 6°C/min	95 mg/L	λpL -cI857	[142]

UVRA: UV resistant-isoform A; IHF: Integration host factor; IFN: interferon; GXP: Guanine-xanthine phosphoribosyltransferase; rpGH: recombinant porcine growth hormone; β-gal: β-galactosidase; SpA: staphylococcal protein; TNF: tumor necrosis factor; TRAIL: TNF-related apoptosis-inducing ligand; GFP: green fluorescent protein; Mut3sY: Shorter version of INF-g gene; rhBMP-2: recombinant human bone morphogenetic protein 2; hFGF-2: human fibroblast growth factor 2; hGH: human growth hormone; scFv: single chain variable fragment; hPPI: Human pre-proinsulin; TK: Thymidine kinase; SEAP alkaline phosphatase; λPL: bacteriophage lambda; IB: Inclusion bodies; TCP: total cell protein; SF: shake flasks; HCDC: high cell density cultures; BC: Batch culture; FB: Fed-batch culture; CC: Continuous culture.

The thermoinduced expression system has been improved throughout the years (Table 1). One of the earliest remarkable advances was the overproduction of the cI repressor by construction of plasmids bearing the lac operon promoter adjacent to the cI repressor gene [56]. Latter on, the first expression vectors using the pL promoter were described and shown to yield recombinant protein at 2.0 to 6.6% of total protein [44,57-59]. Some years latter, recombinant protein yields reached almost 30% of total protein in an *E. coli *strain harboring a pL plasmid and a chromosomal defective prophage with a copy of cI857 [60]. Although Remaut et al. [60] used a similar strategy than Bernard et al. [57], they were able to attain higher production of recombinant protein by decreasing the distance between the pL promoter and the cloned gene. By 1983, an improved plasmid vector that increased productivity was reported [59]. It included the use of temperature-regulated runaway replication that allowed the increase in plasmid copy number and repression release after temperature up-shift. A second improvement, that made possible the use of practically any *E. coli *strain with this system, consisted in the use of a compatible plasmid encoding cI857 [61] (Table 1). Other researchers developed an expression vector that included the pL promoter and the cI857 gene in the same plasmid construction [58]. Several other improvements to the expression vectors have been described, including synthetic ribosome-binding sites and suitable poly-linkers to allow the cloning of any gene to be expressed [62,63]. Recently, mutations in the operator oR have allowed a tight repression even at temperatures as high as 39°C, but still permit heterologous protein expression if temperature is increased between 39°C to 42°C, allowing the use of bacteria, such *Helicobacter*, that are not able to grow at low temperatures [64].

To date, different promoter combinations controlled by cI857 have been reported, including cI857/pR [65,66], cI857/pR/pRM [63], cI857/pL/pR in tandem [10,63,67], and cI857/pL [44,51,58,61,65] (See Table 1). All such combinations appear to work equivalently and all of them share the same advantages, mentioned in the previous section, over other popular expression systems. In addition, the λpL/pR-cI857 expression system shows other important advantages. One single copy of the cI857 gene produces enough repressor to completely inhibit the activity of pL or pR promoters, even if the promoters are present in multicopy plasmids [60]. The thermoinduced system can be used in virtually any *E. coli *strain and even in other Gram-negative bacteria like *Erwinia *and *Serratia *[68]. Furthermore, depending on the protein, bacterial strain, and culture conditions, the use of this expression system can yield as much as 30% of recombinant protein with respect to total cell protein [14].

Some attempts have exploited the strong λ phage promoters and repressor but without employing temperature up-shifts for inducing recombinant protein production. In these cases a wild-type repressor is used instead of the thermolabile cI857 and induction is achieved by adding a chemical inducer such as mitomycin C or nalidixic acid. Such inducers drive heterologous protein expression by triggering the SOS response with the concomitant expression of Rec-A and thus the auto-cleavage of cI [69]. The use of pL promoter has also been proposed for constitutive expression, that is, without cI regulation [10]. This approach can work in the cases where the product is not toxic to the host cell or when plasmid instability (segregational or structural) is not a problem [4]. For example, β-galactosidase has been expressed as a reporter protein under the pL promoter [70]. Interestingly, in such a study it was shown that transcription from pL increased as temperature decreased and that mutations on pL enhanced its activity. Nonetheless, most of the pL/pR expression systems developed use temperature induction to control heterologous protein expression.

Finally, other thermo-regulated expression systems have been developed during the last decade. For instance, thermolabile derivatives of the repressor protein Rro of the temperate *Lactococcus lactis *bacteriophage r1t have been obtained by comparative molecular modeling of the known 3D-structure of cI857 of λ bacteriophage [71]. Another example is the thermo-regulated system conformed by the *lacZop *operator/promotor that is efficiently repressed by the LacIts repressor at 30°C but total induction is achieved upon increasing the temperature to 42°C [72].

## Molecular responses after recombinant protein induction in the thermo-regulated expression system

**Figure 2:**
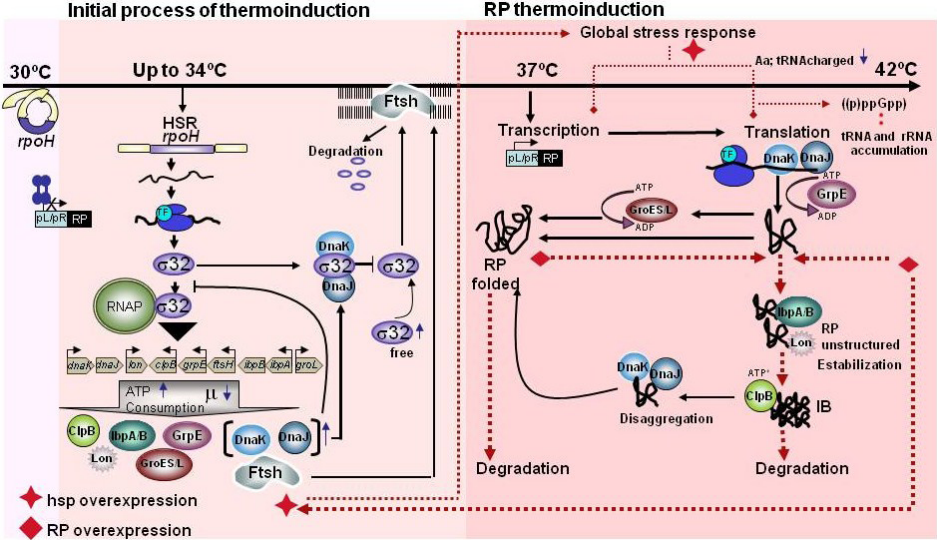
**Schematic diagram of the molecular and physiological responses during thermoinduction**. When cultures are induced upon a temperature increase, σ32 transcription is enhanced. σ32 regulates the expression of hsp, directing the RNA polymerase (RNAP). σ32 activity is controlled by negative feedback through binding to DnaK/J and GroEL/S, whereas it is degraded FtsH. Over-synthesis of hsp initiates the negative feedback control of σ32. Temperature up-shift from 30°C to 34°C initiates the induction of hsp synthesis [23]. Up to 37°C, cI857 repressor is released from the pL/pR promoters and recombinant protein transcription starts. The nascent polypeptides need the assistance of chaperones for correct folding, but some of them remain as unfolded intermediate until their aggregation. Under severe stress conditions, large aggregates can be formed. Reaching 42°C and/or the overproduction of heterologous protein can cause important stresses and metabolic unbalances, generating a global stress response, diminishing growth, and triggering the stringent and the SOS responses. RP: recombinant protein, hsp: heat shock proteins. TF: chaperone trigger factor.

The temperature up-shift required to induce recombinant protein in the thermo-regulated expression system also causes a heat stress that activates the HSR (Figure 2). The HSR initiates with the simultaneous overexpression of orchestrated heat shock genes that protect the cell against thermo-denatured proteins and control homeostasis by increasing the thermotolerance [22,73-75]. In *E. coli*, most of the heat shock genes are transcribed effectively and specifically by the RNA polymerase holoenzyme bound to the alternative σ32 (known also as σH) factor encoded by the *rpoH *gene [76-79]. The σ32 is a master regulator that alters the expression of different genes, including transcription factors, and regulates the activity of the transcriptional apparatus itself as well as executes different roles in cell homeostasis [80]. The σ32 regulon is formed by at least 120 genes organized in hierarchical clusters [80], including almost all coding sequences for proteins involved in folding and degradation, such as chaperones like ClpB, DnaK/J and GroEL/S, and proteases like Lon, ClpP, ClpC, HsIV (ClpY) HsIU, ClpQ, and FtsH [81,82].

Temperature increases may cause nucleotide misincorporation and chromosome damage [80]. However, after σ32 activation, members of the regulon can protect DNA and RNA, maintaining genomic integrity that is essential for cell survival at high temperatures [80]. Also, σ32 regulates the expression of genes coding for proteins that sense glucose and different metallic ions in the extra-cytoplasmic environment, proteins implicated in the secretion and processing of envelope proteins, transporters, proteins related to energy generation, and enzymes necessary for synthesis of the phospholipids, lipopolysaccharides and peptidoglycans needed to maintain cell integrity [80,83]. Other regulon members transfer Δ3-isopentyl-PP to tRNA. Such tRNA modification stabilizes the codon-anticodon pairing and improves tRNA thermal resistance [84,85]. Furthermore, some chaperones participate in the correct function of small RNAs under high temperature [84].

The synthesis level of σ32 is tightly regulated at low temperatures (below 32°C) because its translation start site is occluded by inhibitory base pairing, however, when temperature increases, base pairing is destabilized and *rpoH *translation is enhanced [86,87]. At 30°C, about 50 molecules of σ32 per cell exist, whereas such amount increases about 17-fold shortly (5 to 6 min) after temperature is up-shifted to 42°C, and decreases back to close to 250 molecules per cell 15 min after the temperature up-shift [88]. It has been shown that increasing the temperature to 37°C activates translation of *rpoH *transcripts [88,89]. The activity of σ32 is regulated by the so called "negative feedback loop" whereby chaperones such as DnaK/J and GroEL/S interact with σ32, limiting its binding to RNA polymerase [25,90,91] (Figure 2). When unfolded proteins are high relative to DnaK, this protein is titrated away from σ32, triggering the HSR [25]. In contrast, when unfolded proteins are scarce, the DnaK/J and GroEL/S systems mediate the degradation of σ32 [25,92]. It has been suggested that DnaK can help degrade σ32 by delivering it to the protease FtsH [92]. The current model for σ32 regulation by DnaK chaperone has been referred to as the "unfolded protein titration" [73,93].

In addition to the cellular stress caused by an increase in temperature, protein overexpression by itself can also trigger stress responses. Recombinant protein produced in the thermoinduced system can reach high concentrations and accumulate as inclusion bodies [7,15,94,95]. It is well known that overexpression and accumulation of unfolded recombinant proteins direct the response of genes involved in protein folding and degradation, which in turn are mainly controlled by σ32 [17,96,97]. The molecular responses observed include the upregulation of genes coding for heat shock proteins like DnaK, DnaJ, GroEL, GroES, ClpP, GrpE, DegP, IbpA, IbpB, and FtsH [17,97], as well as the genes *ompT *and *ftsH*, that code for proteases [17]. Moreover, the upregulation of genes involved in the SOS response, such as *recA *or *lon*, has also been reported [17,97]. It must be emphasized that activation of the molecular response to unfolded protein accumulation, as well as its duration, are dependent on the recombinant protein produced [17].

The molecular responses activated by heat or by unfolded protein accumulation have been studied extensively in separate contexts [17,80,97-99]. However, both responses converge in the activation of genes coding for chaperones and proteases. For instance, some genes (such as *dnaK*, *dnaJ*, *ibpA*, *ibpB*, *lon*, *ftsH clpB*, *rpoS*, *rpoH*, *ompT*, and *groEL*) are rapidly upregulated within the first few minutes after heat shock or recombinant protein accumulation. In particular, *dnaK*, *dnaJ, clpB *and *ibpA *increase several times during the initial 5 min [15,17,80,88], while, the heterologous gene can increase as much as 150 times in 2 min [15]. Upregulation in general does not last for more than 40 min. In contrast, the upregulation of *lon*, *groEL*, and *ompT*, in response to accumulation of proteins like interleukin 2 (IL-2) or viral protein (VP5), can be maintained for more than 40 min [17].

Recently, transcriptomic and transcriptional approaches to analyze cultures with dual stress, consisting of heating above 37°C and inducing the accumulation of unfolded recombinant protein, have been performed [15,100]. Although, different protocols of heat and induction have been applied, both studies show that the dual stress activates genes like *rpoH *and those associated with the heat shock response (*dnaJ, dnaK*, *htpG*, *groEL*, and *groES*) [15,100]. In a transcriptomic study by Harcum and Haddadin [100], the molecular responses in cultures heated at 50°C, cultures induced chemically with IPTG, and cultures with dual stress were analyzed. They found that 163 genes from 1,881 studied, responded strongly in cultures with dual stress compared with cultures only heated or induced [100]. In particular, genes coding for RNA polymerase like *rpoA/S *and ribosome coding genes were downregulated, meaning that transcription and translation probably diminished. Moreover, genes associated to the pyruvate metabolism and glutamine biosynthesis were also downregulated. Interestingly, *relA *was downregulated [100]. Such gene codes for the protein guanosine 5'-triphosphate 3'diphosphate or guanosine 3',5'biphosphate (p)ppGpp synthetase I, which participates in the stringent response and its activation has been observed in response to protein production and accumulation [96]. In another study, a transcriptional analysis was performed to understand the cellular response to heat and overproduction of heterologous protein using the λpL-cI857 thermoinduced system [15]. In this case, temperature rather than chemical induction was employed. The results showed that heat shock proteins and proteases were upregulated several times, while the transcriptional levels of critical genes that control the heat-shock response (*rpoH *and *ftsH*) had a relatively small increase (0.5 times) [15]. Furthermore, genes corresponding to the general stress response (*rpoS*), and those that code for RelA and SpoT (the proteins that control the stringent response) showed no significant changes.

## Physiological responses after induction in the thermo-regulated system

One of the most notable changes in cell physiology upon induction by temperature up-shift is the decrease in specific growth rate, which is inversely related to recombinant protein synthesis rate [21]. Other changes include the increase in respiration [75], alteration of central carbon metabolism [75,101-103], modification of the lipid to protein ratio in the membranes [26,80], ribosome destruction and DNA relaxation [104]. In particular, after increasing the temperature, the global protein synthesis increases by a factor of three, of which 20-25% correspond to hsp [22,23,99,105,106]. Such an increase generates an unstable environment that causes a critical metabolic burden that impacts growth rate and quantity of the protein produced [99,106,107]. The high rate of protein synthesis can also exhaust carbon skeletons and amino acids pools, mainly when minimal media is used [108,109]. Depletion of amino acids can result in large pools of deacylated tRNAs that upon attachment to ribosomes are recognized by RelA. This in turn triggers the immediate utilization of ATP and GTP or GDP by RelA to synthesize AMP and (p)ppGpp [110-112]. The (p)ppGpp alarmones activate the stringent stress response and promote a higher transcription of heat-shock and other stress related genes such as those that code for proteases, which mainly degrade ribosomes [19,113,114]. During the stringent stress response, the synthesis of tRNA and rRNA, as well as the transcription of genes from the transcriptional-translational cell machinery are downregulated and thus the translation process is interrupted. This limits protein synthesis and cell growth capacities during recombinant protein production [19,101,109,115].

Activation of heterologous protein overexpression and endogenous hsp, in response to thermoinduction, also increase the requirements for ATP by a factor of six [75,116]. Such a huge energy requirement is responsible for the observed increase in respiration, but is not enough to compensate the ATP demand for protein production, causing a transient drop in the cell energy charge [75,103,117].

Under non-limiting glucose concentration, a better glucose assimilation is ensured by overexpression of genes coding for cAMP-catabolite regulation protein (CRP), and repression of genes coding for TCA enzymes. Also, high ATP requirement stimulates carbon flux through glycolysis followed by a decrease in growth rate [118]. For example, Wittmann et al. [103] reported that these entire metabolic changes impaired specific growth rate from 0.45 h^-1 ^before induction, to only 0.17 h^-1 ^after expression of the recombinant gene. Specifically, a temperature up-shift stimulated more than 20% of the glycolytic flux, whereas it decreased carbon fluxes through pentose phosphate and other biosynthetic pathways by around 57%. In addition, fluxes around TCA cycle dropped by 35%, but activation of the glyoxylate shunt was observed [103]. Moreover, after induction carbon flux through the pyruvate node is preferentially channeled through pyruvate oxidase, resulting in acetate accumulation [103,119]. Accumulation of organic acids has been observed during the induction phase as a means to compensate the unbalance at the pyruvate node due to the reduction in the flux through pyruvate dehydrogenase and the TCA cycle [15,120].

When glucose is the limiting nutrient, carbon fluxes through the pentose phosphate pathway decrease upon temperature up-shift, whereas the fluxes through the Embden-Meyerhof-Parnas and TCA cycle increase, leading to a reduced flux through growth-associated pathways, such as the anabolic pathways [102]. Also, the anaplerotic reactions operate at low levels [102]. As the TCA activity is increased, 75% of the carbon source is converted into CO_2 _in induced cultures, compared to only 25% in cultures at 30°C [75,102,103]. Consequently, carbon flux to acetate formation is absent during induced cultures under glucose limitation [103,121,122].

The metabolic and physiologic differences between induced and non-induced cultures impact glucose consumption and difficult the establishment of glucose feeding strategies during the post-induction phase. For instance, although the most common strategy to avoid overflow metabolism during induction is to reduce the pre-induction glucose-feeding rate, such an approach can result in carbon source limitation that reduces recombinant protein production [109,123], and activates ppGpp synthesis and the stringent stress response [101].

## Phenomena and variables that modulate the productivity in the thermo-regulated system

Several phenomena and variables, that can modify or negatively affect recombinant protein productivity of the λpL/pR-cI857 system, must be considered for optimal bioprocess performance. Among them, the most important include plasmid segregation, host strain, fermentation process, heating strategies (such as time and temperature of induction), cellular site where the recombinant protein is localized, and even the type of recombinant protein expressed [55,124,125]. The yields reported in the literature using the λpL or pR/cI857 thermoinduced system with different plasmids, under different culture and induction conditions, and expressing different recombinant protein are summarized in Table 1. Data have been grouped depending on the cellular location where the recombinant proteins accumulate.

### Plasmid segregation

It has been shown that during prolonged periods of derepression of the λpL/pR promoters at temperatures up to 42°C, the propagation of plasmid-free cells is favored [16,126,127]. Furthermore, the fraction of plasmid-free cells under derepression at 38 or 40°C has been reported to be lower than at 41°C or above [16,127]. For example, Sayadi et al. [128] detected that 12 and 48% of the cells had lost the plasmid after 250 generations at 37 and 42°C, respectively. Nonetheless, some plasmids such as the thermoinduced pCY-TNF, show high segregational stability, as 70% of cells still harbor the plasmid after 200 generations in cultures at 42°C without selection pressure [10].

Plasmid instability during temperature up-shifts generally occurs in the pL/pR promoter system [52,129] because in dividing cells a partition mechanism necessary for stable plasmid inheritance has usually not been incorporated [128,130-132]. Examples of such mechanisms in other expression systems include bacterial plasmids encoding for the partitioning (*par*) loci, that ensure ordered plasmid segregation to daughter cells during division [133]. Moreover, plasmid maintenance and replication in host cells cause a metabolic load and the consumption of important cell resources [4,134]. Such a metabolic burden and energetic drain further increases upon induction of heterologous protein [4,135]. It has been shown that the plasmid load causes a decrease in cell specific growth rate compared to plasmid-free cells [128,130-132].

Different operational conditions have been proposed to avoid plasmid segregation and extend the production phase after induction. For instance, plasmid copy number can be maintained by restricting the specific growth rate to low values [136,137]. This can be achieved through fed-batch protocols that result in high cell concentrations, such as in cultures with linearly or exponentially increasing rates of substrate addition before induction [7,54,129]. Another option, although rarely reported, is the use of two continuous cultures connected in series [128]. Cells are initially grown in a first chemostat at temperatures low enough to keep the promoter repressed and to conserve plasmid copy number. The effluent of the first chemostat is continuously fed to a second bioreactor, maintained at a higher temperature, where production of the recombinant protein occurs [128]. In such two-component systems high plasmid stability and high cell density cultures can be obtained in the first tank, whereas high productivity is achieved in the thermally induced bioreactor.

### Host strain

Different *E. coli *strains present different heterologous gene expression capacities [138,139], but few studies have attempted to determine the effect of host strain on recombinant protein production using plasmids that contain λ elements. In one of such studies, different strains were transformed with plasmid λPL-DsbA (peptide signal of the periplasmic protein DsbA) containing the coding *hGH *gene [125]. The highest hGH expression was obtained in W3110 and RB791 strains (19.6 and 16.2% of hGH with respect to total mass, respectively), whereas other strains produced several-fold less. One factor that may be important to consider for increasing the productivity is the use of protease-deficient strains [140]. For instance, Choi and Lee [141] reported that in a non-thermally induced culture, the protease-deficient BL21 *E. coli *was the most productive strain.

### Recombinant protein

As described previously, the expression of a heterologous protein in the thermoinduced system triggers specific molecular and physiological responses in the host cell that can ultimately degrade the recombinant protein. However, the nature of each protein will be the main determinant of its stability [17]. Nonetheless, few reports have attempted to understand the effect of the particular recombinant protein being expressed by maintaining the same thermoinduced plasmid, host strain, and culture conditions. Even scarcer are the reports that dissect the particular effect of the recombinant protein on the molecular or physiological responses using the same expression system. An exception is the work by Soares et al. [142] who compared the expression of hGH and human prolactine (hPRL) using the same heat-inducible system. The hGH was successfully expressed and secreted into the *E. coli *periplasm, with yields above 1 mgL^-1^A_600_^-1^, whereas the yields for hPRL were about 50-times lower. Soares et al. [142] suggested that such an important difference in production between both proteins could be caused by differences in the thermal lability and/or proteolytic sensitivity of the heterologous proteins.

### Culture strategies

Different culture strategies have been investigated to search for the optimum production scheme. Strategies such as the addition of complex-rich media with yeast extract, peptone or tryptone, alone or mixed [143], and protease inhibitors [143,144] are commonly used. Other factors that can be controlled to improve the productivity are the induction temperature, duration of the induction, and the specific growth rate before or after induction [52,136]. Different culture modes, including batch, fed-batch and continuous, have been used to investigate the relationship between specific growth rate and protein production. Fed-batch processes have been particularly exploited for such purpose as these allow a tight control of the growth phase and can be temporally separated from the production phase, while maintaining plasmid stability and avoiding metabolic stress and production of toxic organic acids [52,94,136,137,145]. For example, by controlling the substrate feed rate during the growth phase and the specific growth rate during the production phase, Lim and Jung [52] attained high cell densities and a 23-fold increase in final interferon-γ concentration in comparison with batch cultures. Likewise, Curless et al. [136] produced interferon-α by initially culturing the cells in a chemostat at 30°C under glucose limitation; after a steady state was achieved, a fed-batch mode was initiated and the temperature increased to 42°C. Production of interferon-α increased 4-fold under the higher dilution rates tested, demonstrating the dependence of the pre-induction specific growth rate on productivity. As detailed previously, continuous cultures can also be employed with the thermoinduced expression system as long as the growth phase is separated from the production phase by means of two serially connected chemostats [126,128].

### Heating strategies

A variety of heating strategies have been developed to induce the expression of recombinant proteins. The main aims of such strategies have been to avoid the adverse effects of high temperature, such as decreased growth rate, damage to the host cells, decrease in viability and productivity, and plasmid instability [15,54,55,95,115,129,137,142,146,147]. Frequently, the temperature is increased from 28-32°C to 40-42°C. In some cases, a few minutes after the temperature has been raised, it is decreased back to 38°C or 40°C (Table 1). For example, Tabandeh et al. [54] induced cultures at 42°C for 20 or 40 min and then decreased the temperature to 37°C for 4 h. They reported that the recombinant protein was degraded when the induction phase at 42°C lasted 40 min, whereas degradation was absent if temperature was decreased to 37°C within 20 minutes after induction.

The heating rate in a bioreactor is a relevant parameter because it can substantially differ from laboratory to large-scale, due to the fact that the ratio between the heat transfer area to volume decreases in an inverse function to the size of conventional stirred-tank bioreactors [148]. Nonetheless, such a parameter was overlooked until the recent work by Caspeta et al. [15], who established a scale-down approach to understand how heating rate differences, related to scale, affected recombinant protein production and cell performance. In such a study, heating rates of 6, 1.7, 0.8, and 0.4°C/min, typical of 0.1, 5, 20, and 100 m^3 ^fermentors, respectively, were simulated in a laboratory scale bioreactor [15]. The maximum recombinant protein production and minimum accumulation of waste organic acid by-products was obtained during the slowest heating rates that emulated the largest scale fermentors [15]. Such results demonstrated that during faster heating rates, typical of laboratory conditions, the cells required more energy and experienced larger imbalances between glycolysis and the TCA cycle than during slower heating rates characteristic of large-scale vessels. The study also demonstrated that cells subjected to slow heating rates can better adapt to thermal stresses than those exposed to a faster temperature increase [15].

### Protein accumulation and recovery

Recombinant proteins produced in the thermoinduced λpL/pR-cI857 expression system can form aggregates in the cytoplasm, accumulate in soluble form either in the cytoplasm or the periplasmic space, or be secreted to the supernatant [7,65,125,149]. The site of protein accumulation depends on proper localization signals [125]. Production of recombinant proteins in the periplasmic space offers several advantages, such as decreased proteolytic activity compared to the cytoplasm, simpler purification protocols as fewer host proteins are present in the periplasm, and minimum amounts or even absence of undesirable isoforms and posttranscriptional modifications. Furthermore, the in vivo cleavage of the signal peptide provides authentic N-terminus of the target protein (i.e., without an extra methionine). Another advantage is that correct formation of disulfide bonds can be facilitated because the periplasmic space provides a more oxidative environment than the cytoplasm [14,125]. Until now, few examples of soluble recombinant protein expression in periplasm using the λpL/pR-cI857 system have been reported [125,142].

The increased temperature and high production rates alter protein folding, which in turn favors protein aggregation into inclusion bodies (IB) when using the λpL/pR-cI857 system [65,137,149,150]. The factors that determine IB formation include the nature of the protein expressed (v. gr., protein sequence), growth and induction conditions, cytoplasmic reducing environment, kinetic competition between aggregation and folding, high local protein concentration, inappropriate interactions with *in vivo *folding chaperones, intermolecular disulfide crosslinking (although proteins lacking cysteines can still form inclusion bodies), medium composition, and bacterial strain [137,150-153]. Accumulation of the recombinant protein in IB has certain advantages, as it can be isolated and concentrated by a simple centrifugation step, reducing the downstream processing costs and facilitating the production of toxic proteins to cells [154-156]. However, a refolding step is then required to recover a biologically active protein, and refolding steps can be inefficient [157,158].

Induction temperature, extracellular pH, and the carbon source can either prevent or accelerate the formation of IB when employing the λpL/pR-cI857 system [151]. IB typically contain around 20% to 85% of recombinant protein, whereas the remainder consists of a large set of host cellular proteins, particularly hsp such as chaperones and co-chaperones [153,155,159,160], and other contaminants such as phospholipids, nucleic acids, and membrane proteins [152]. The chaperones DnaK and GroEL (together with their co-chaperones DnaJ-GrpE and GroES, respectively) have been identified as major components of IB produced in the thermoinduced system [155]. DnaK has been mostly localized on the surface of the IB, suggesting that this protein interacts with the IB after their formation [155]. In contrast, a significant fraction of GroEL has been observed within the IB, remaining trapped inside during the initial aggregation [155]. Interestingly, the absence of DnaK and GroEL in *E. coli *mutants significantly reduces bacterial growth [161]. Absence of DnaK results in larger IB, whereas a deficiency of GroEL reduces aggregation and favors soluble protein formation, with the concomitant production of smaller and numerous IB [155]. This has been explained as a combined consequence of a less efficient folding and impaired rescue (removal) of aggregates [161].

Interestingly, expression systems that are not based on heat induction have exploited phenomena present in the heat inducible expression system to improve various aspects of recombinant protein production. For instance, chaperones like DnaK/J or DnaK/J-GrpE/ClpB have been co-expressed to facilitate disaggregation and refolding of the recombinant protein of interest [162,163]. Likewise, the co-expression of GroEL/S with heterologous proteins such as zeta-crystallin [164] and carbamoylase [165] has significantly enhanced the yield of soluble proteins. Other examples include the co-expression of the DnaK-DnaJ-GrpE chaperones to increase the solubility of endostatin and human ORP150, or the overexpression of DnaJ to increase soluble transglutaminase content [166,167]. Overexpression of all such chaperons naturally occurs during thermoinduction of heterologous protein. Accordingly, the knowledge gained on the molecular and physiological events that occur during recombinant protein production by the λpL/pR-cI857 system can be of great value to control and improve not only this expression system but others as well.

## Concluding remarks and future directions

Many expression systems have been developed to increase recombinant protein production in *E. coli*, yet, the thermo-regulated system is particularly appropriate for large-scale protein production, because it is highly productive, can be finely regulated, is easily scalable, minimizes culture handling, and avoids the use of chemical inducers. However, there are still several unresolved problems, related to the molecular and physiological responses during induction of recombinant proteins, which cause an increased energy demand and metabolic burden that affect the final productivity and product quality. Other challenges, yet to be tackled, include the better exploitation of the beneficial effects of activation of σ32 regulon members during thermoinduction, while avoiding the detrimental consequences. For instance, while overexpression of chaperones, proteases, and other proteins during thermoinduction can prevent protein aggregation and protect DNA and RNA that are necessary to maintain cellular integrity, it also represents an important drain in energy and precursors that are diverted from the production of the protein of interest. Important molecular responses occur quickly within the first two minutes after thermoinduction of heterologous protein and can last for more than 40 minutes. A better understanding of the kinetic behavior of such responses, for instance by transcriptional and proteomic approaches, will yield an integral knowledge of the phenomena that should allow the design of novel regulation and control strategies for improving recombinant protein production.

With the aim of improving productivity, the recombinant proteins expressed under the thermoinduced system have been produced under different culture conditions, heating protocols, and recovery strategies, that can result in very different yields [3,7,168]. The development of novel induction strategies is an avenue of great potential. It is desirable to design protocols that avoid growth cessation, increase productivity and improve the purification of the recombinant protein, by modulating molecular responses such as the HSR. For instance, oscillatory temperature induction protocols could be exploited for such purpose. In this regard, there still remain questions related to the molecular and physiological responses, in particular, when non-conventional heating protocols are used. Finally, recovery and *in vitro *folding of heterologous proteins is a complex task due to the presence of various contaminant compounds. An interesting field that must be explored is the manipulation of the induction conditions to minimize the accumulation of impurities and favor the production of specific chaperones in order to improve product recovery and folding. Specifically a proteomic analysis of IB should help to identify contaminants that reduce yield or difficult the purification.

## Competing interests

The authors declare that they have no competing interests.

## Authors' contributions

NAVC, OTR, NOP and MATR suggested and defined the topic of this review article based on their industrial experience working with this particular expression system and their academic experience working with other expression systems. NAVC drafted the manuscript, OTR, MATR, NOP and LC revised it critically and amended the manuscript. All authors read and approved the final manuscript.
